# Distinct astrocyte phenotypes aid microglia‐mediated Aβ clearance in immunized Alzheimer's disease patients

**DOI:** 10.1002/alz70855_100543

**Published:** 2025-12-23

**Authors:** Alex J Edwards, Lily Camp, Anne V Forsyth, Joshua Kuruvilla, Brooke Simonton, Pouya Jamshidi, Rudolph J Castellani, James AR Nicoll, Delphine Boche, Lynn van Olst, David Gate

**Affiliations:** ^1^ Northwestern University Feinberg School of Medicine, Chicago, IL, USA; ^2^ Abrams Research Center on Neurogenomics, Chicago, IL, USA; ^3^ Mesulam Center for Cognitive Neurology & Alzheimer's Disease, Chicago, IL, USA; ^4^ University of Southampton, Southampton, Hampshire, United Kingdom

## Abstract

**Background:**

Recently approved Alzheimer's disease (AD) therapies leverage immunization strategies targeting amyloid‐β (Aβ). While most studies focus on microglial responses to these treatments, evidence suggests that sustained immune activity and Aβ clearance drive broader cellular changes. Astrocytes, which accumulate at microglia‐targeted Aβ plaques, may play a complementary role in clearance. This study examines how astrocyte phenotypes are altered by Aβ immunization and their contribution to Aβ clearance.

**Methods:**

We performed single‐cell RNA sequencing and spatial transcriptomics on post‐mortem brain tissue from 16 AD patients—10 immunized (9 with AN1792 from the first active Aβ immunotherapy trial, 1 with lecanemab) and 6 non‐immunized controls. We identified astrocyte states enriched in immunized brains and analyzed their transcriptomic profiles. Additionally, we mapped astrocyte states localized at microglia‐targeted Aβ plaques.

**Results:**

A distinct astrocyte population emerged following immunization with both AN1792 and lecanemab, characterized by upregulated *CHI3L1* and other reactive astrocyte markers. Spatial analysis revealed these astrocytes preferentially localize at microglia‐targeted Aβ plaques post‐lecanemab treatment. Ongoing analyses aim to further define astrocyte phenotypes in terms of morphology, protein expression, Aβ uptake, and spatial relationships with Aβ pathology and microglia.

**Conclusion:**

We identify a unique astrocyte phenotype induced by Aβ immunization in AD, marked by high *CHI3L1* expression. This astrocyte state may be driven by microglia at Aβ plaques and contribute to plaque clearance. Our findings highlight distinct astrocyte responses to Aβ immunization, shedding light on their role in therapeutic outcomes.

